# Model-based characterization of total serum bilirubin dynamics in preterm infants

**DOI:** 10.1038/s41390-024-03644-z

**Published:** 2024-11-07

**Authors:** Meng Chen, Alain Beuchée, Emmanuelle Levine, Laurent Storme, Geraldine Gascoin, Alfredo I. Hernández

**Affiliations:** 1 University of Rennes, Rennes University Hospital, LTSI-INSERM U1099, F-35000 Rennes, France; 2 Department of Neonatology, Lille University Hospital, F-59000 Lille, France; 3 Department of Neonatology, Angers University Hospital, F-49000 Angers, France

## Abstract

**Objectives:**

This study aims to characterize the age-related natural dynamics of total serum bilirubin (TSB) in preterm infants through a mathematical model and to study the model parameters as potential biomarkers for detecting associated morbidities.

**Methods:**

We proposed an exponential decay model and applied it to each infant. Patient-specific parameters were obtained by minimizing the error between measured TSB and model output. Modeling evaluation was based on root-mean-square error (RMSE). The occurrence of high-risk clinical events was analyzed based on RMSE.

**Results:**

In a subset of the CARESS-Premi study involving 373 preterm infants (24–32 weeks’ gestation), 72 patient-specific models were fitted. RMSE ranged from 1.20 to 40.25 µmol/L, with a median [IQR] of 8.74 [4.89, 14.25] µmol/L.

**Conclusions:**

Our model effectively characterized TSB dynamics for 72 patients, providing valuable insights from model parameters and fitting errors. To our knowledge, this is the first long-term mathematical description of natural TSB decay in preterm infants. Furthermore, the model was able to estimate the occurrence of clinical events such as necrotizing enterocolitis, as reflected by the relatively high RMSE. Future implications include the development of model-based clinical decision support systems for optimizing NICU monitoring and detecting high-risk events.

**Impact:**

- The study characterizes the natural dynamics of total serum bilirubin in preterm infants (24–32 weeks’ gestation) using a patient-specific exponential decay model.
- The model describes patient-specific patterns of TSB evolution from day three to the first weeks, providing a median [IQR] root-mean-squared error of 8.74 [4.89, 14.25] µmol/L.
- Complementary to previous studies focusing on the first 72–96 h, our study emphasizes the later decay course, contributing to a comprehensive long-term characterization of the natural TSB dynamics in preterm infants.
- The proposed model holds potential for clinical decision support systems for the optimization of NICU monitoring and high-risk event detection.

## Introduction

Hyperbilirubinemia affects 60–80% of newborns.^1^ Elevated unconjugated bilirubin induces neurologic dysfunction by depositing in selective brain regions including the basal ganglia, hippocampus, and brainstem nuclei.^2^ Hyperbilirubinemia can manifest as acute bilirubin encephalopathy (ABE) shortly after birth, presenting with non-specific clinical manifestations such as lethargy, hypotonia, poor feeding, recurrent apnea, and desaturations.^3–5^ Chronic bilirubin encephalopathy (CBE) may ensue, known as kernicterus, causing permanent neurologic sequelae such as athetoid palsy and dystonia, hearing loss, and oculomotor impairments.^3^ Particularly, premature infants born before 32 weeks of gestation are especially vulnerable to the neurotoxic effects of hyperbilirubinemia, which can be more severe and long-lasting.

The management of neonatal hyperbilirubinemia is guided by protocols from organizations such as the American Academy of Pediatrics (AAP)^6^ and the National Institute for Health and Care Excellence (NICE),^1^ with adaptions in national standards across different countries and regions.^7–11^ Research efforts have primarily focused on the early postnatal evolution of total serum bilirubin (TSB) within the first 72 or 96 h of life,^12–15^ with less attention given to the prolonged course in preterm (28–32 weeks of gestation) and extremely preterm infants (less than 28 weeks of gestation), who are more likely to face potentially life-threatening conditions.

The ability of preterm infants to metabolize and excrete bilirubin matures as aging after birth. After the period of physiological neonatal jaundice (usually one week after birth), as the liver matures, its ability to metabolize bilirubin improves, leading to a progressive reduction in the accumulation of bilirubin in the blood. An important aspect of the metabolic pathway of bilirubin excretion is the bilirubin conjugating capacity of the liver, mediated by hepatic bilirubin UDP-glucuronosyltransferase activity.^16^ This enzyme activity is less than 1% of adult values during the fetal and early neonatal period and then begins to increase at an exponential rate until it reaches the adult value by 14 weeks of age, after which it remains constant.^17^

Therefore, in this study, we focus on a population of preterm infants and hypothesize that the natural dynamics of TSB concentrations from three postnatal days could be quantified and modeled through an exponential decay. Furthermore, the parameters of such a model, once fitted to reflect the TSB dynamics at the individual level, might be used to characterize the maturation and functionality of the bilirubin conjugation pathway. The primary objective of this study is to investigate the characteristics of the age-related dynamics of TSB levels during the early postnatal period, aiming to track and anticipate developmental patterns in this population. Our secondary objective is to validate the model’s parameters as potential biomarkers for detecting relevant comorbidities when bilirubin evolutionary trends diverge from expected decays.

## Materials and Methods

### Study population and inclusion criteria

A prospective multi-center clinical study CARESS-Premi (NCT01611740) was conducted with the main objective of developing a computer-assisted diagnostic tool based on real-time analysis of cardio-respiratory signals to help neonatologists in the diagnosis of infection at the bedside of preterm infants. Within the scope of this initiative, we performed an ancillary study to estimate TSB levels using general clinical characteristics and longitudinal cardio-respiratory data. The cohort prospectively involved 519 preterm infants born between 24 and 32 weeks of gestational age (GA) with birth weights > 500 g, hospitalized in the neonatal intensive care units (NICU) across three university hospitals in France (University Hospitals of Rennes, Angers and Lille) between October 2012 and November 2018. The dominant center – University Hospital of Rennes (CHU) – consisting of 414 patients – is considered in this work. Inclusion and follow-up took place when postnatal age (PNA) > 72 h and postmenstrual age (PMA) < 34 weeks. The protocol was conducted with approval by the local ethics committee and informed parental consent was obtained.

Within the considered group, 373 infants underwent bilirubin-related conditions during the follow-ups, and they were therefore eligible for analysis. Next, infants with fewer than 4 measurements were excluded in the first stage due to the limitation in the minimum number of samples required to fit a reasonably robust model. Then, we examined three conditions within the included population and considered them for exclusion. The first category comprises patients with infrequent monitoring, and those with adjacent TSB measurements taken more than 10 days apart. This exclusion was necessary since sparse sampling might fail to capture possible intermediate abnormal fluctuations in TSB levels, leading to inaccurate or overly optimistic representation of the bilirubin decay in the model fitting process. The other two categories are exchange transfusion (ET) recipients under NICE guidelines, and phototherapy (PT) recipients documented in the database. Patients exhibiting infrequent TSB monitoring were directly excluded from the population. For ET and PT recipients, patients with at least 4 measurements after removal of samples collected during treatment were retained for further analysis. These exclusions ensure that the models effectively characterize the natural evolution of TSB levels, unaffected by treatments that may alter TSB dynamics.

No blood sample was specifically collected for the study; instead, blood samples used to determine total serum bilirubin levels were collected for routine clinical care and adhered to standard care criteria. In addition to the PNA and TSB measurements, relevant clinical characteristics including demographic, maternal, laboratory tests such as C-reactive protein (CRP), treatments, and short-term outcome data were extracted from the CARESS-Premi database. Necrotizing enterocolitis (NEC) was defined as a grade II-b or higher according to the modified Bell’s staging criteria.^18^ The Z-scored birth weights based on gestational age are additionally calculated according to the sex-specific Fenton 2013 preterm growth charts which were developed by meta-analyses of about 4 million births from six countries.^19^

### General TSB decay models

An overall physiological downward trend in total serum bilirubin (TSB) levels after birth, following physiological neonatal jaundice and some fluctuations due to various events, is observed among premature newborns.^16,17,20^ To characterize the decay trend, we first compared two general models: a linear regression model and a basic exponential decay model. These mathematical models were fitted to all available samples from all relevant infants, sorted by postnatal age (PNA), without accounting for individual variability.

A simple linear regression model assumes that the TSB levels decrease linearly over time, expressed as:

1 $${{\boldsymbol{TSB}}}\left(t\right)=A+B{{\boldsymbol{t}}}\,{{\boldsymbol{+}}}\,{{\rm{\epsilon }}}$$

where $t$ is the PNA in days when the corresponding TSB was determined, $A$ is the intercept, $B$ is the slope, and $\epsilon$ is the error term capturing deviations of the fitted line from samples.

The basic exponential decay model posits that the TSB levels decline exponentially, with the rate of decay proportional to the current value of TSB, formulated as:

2 $${{{\boldsymbol{TSB}}}}\left(t\right)=A\times {e}^{-B{{{\boldsymbol{t}}}}}+{{{\rm{C}}}}+{{{\rm{\epsilon }}}}$$

where $A$ represents the initial TSB level, $B$ is the decay factor indicating the rate of decline, $C$ is the constant term representing the baseline level that TSB asymptotically approaches, and $\epsilon$ is the error term. Note that the negative sign before parameter $B$ guarantees the decay of the function.

### Patient-specific TSB exponential decay model

Recognizing the need to accommodate the inter-individual variability in TSB dynamics among premature infants, we further developed a patient-specific exponential decay model. This model, namely,  accounts for individual differences by fitting the TSB evolution trend for each infant individually and includes adjustments based on their gestational age (GA), namely, $g\left(\cdot \right)$. For each subject $i$, the patient-specific exponential decay model is formulated as:

3 $${{{\boldsymbol{y}}}}_{i}(t)=g\left({{{\boldsymbol{p}}}}_{i}(t);{A}_{i},{B}_{i},{C}_{i},{G{A}_{i},Tc}_{i}\right)+{{{\boldsymbol{\epsilon }}}}_{i}={A}_{i}\times {e}^{-{B}_{i}[{{{\boldsymbol{p}}}}_{i}\left({{\boldsymbol{t}}}\right)+G{A}_{i}+{{Tc}}_{i}]}+{C}_{i}+{{{\boldsymbol{\epsilon }}}}_{i}$$

where ${{{{\boldsymbol{p}}}}}_{i}(t)$ refers to a sequence of PNA (days) as the independent variable, ${{{{\boldsymbol{y}}}}}_{i}(t)$ is the TSB concentrations (µmol/L) observed at ${{{{\boldsymbol{p}}}}}_{i}$ corresponding time ${{{\boldsymbol{t}}}}$ as the dependent variable, and ${{{{\boldsymbol{\epsilon }}}}}_{i}$ is the associated modeling error. The parameters ${A}_{i}$, ${B}_{i}$ and ${C}_{i}$ are specific to each infant ($i$), determining the curve shape and how it evolves over time. Additionally, to personalize the modeling of TSB dynamics across patients, we introduced two other factors acting as time-shifting terms: $G{A}_{i}$ and ${{Tc}}_{i}$, denoting the GA at birth (days) and the correction factor, respectively.

We assigned four parameters (${A}_{i}$, ${B}_{i}$, ${C}_{i}$ and ${{Tc}}_{i}$) using a robust non-linear least squared method^21,22^ in a patient-specific manner. The limits of the parameters were set as ***A*** ∈ [0, 200], ***B*** ∈ [0, 1.5], **C** ∈ [0, +*∞*) and ***Tc*** ∈ (−∞, +∞). These parameters were iteratively adjusted within the specified boundaries throughout the fitting process to minimize the sum of squared residuals between the estimated and observed TSB values. To balance the influence of different samples, a smooth approximation of LASSO regression was configured, and an adaptive robustness strategy was also devised to flexibly adjust the optimization process, enabling better adaptation to the physiological characteristics of different subjects. Finally, the parameter set that achieved the lowest sum of squared residuals was assigned as the model parameters for a given patient.

### Model analyses: from patient-specific models to clinical outcomes

Further analyses were conducted after developing patient-specific TSB exponential decay models. First, the distribution of the model parameters was examined. Histograms were created to visualize the range and central tendencies of these parameters across the modeling population, providing insights into the variability, consistency and their contributions to overall model performance. Next, we explored the association between the RMSE of the models and the occurrence of high-risk clinical events, such as NEC and CRP levels, aiming to determine whether the models were indicative of underlying clinical complications. Finally, a median model was constructed using the median values of the parameters from patient-specific models. A local sensitivity analysis was performed on this median model to investigate the impact of individual parameters on the model trajectory. This was implemented by varying each parameter one-at-a-time within its defined range and observing the effects on the model.

### Statistical analyses

The statistics of patient characteristics were reported as “counts (percentage)” for categorical variables and as “mean (SD); median” or “median [IQR]” for continuous variables, as appropriate. Categorical variables were compared using the chi-square test and continuous variables using the Mann-Whitney U test, as appropriate. The significance level was set to 0.05 and adjusted by the Bonferroni correction, thus in this case, *p*-value < 0.0025 was considered significant.

The correlation between TSB in µmol/L and PNA in days was assessed using Pearson’s correlation coefficient. The linear regression was conducted using an ordinary least-squared method. The general exponential decay model was fitted using a non-linear least-squared method. Patient-specific exponential models were fitted using a robust non-linear least squared method with an adaptive strategy mediating the robustness in the optimization process. The fitting error was evaluated by root-mean-square error (RMSE) of the estimated and observed TSB levels.

All calculations and graphics were performed and generated in Python (version 3.8) using Spyder IDE software (version 5.4.3).

## Results

### Population and TSB measurements

**Fig. 1:**
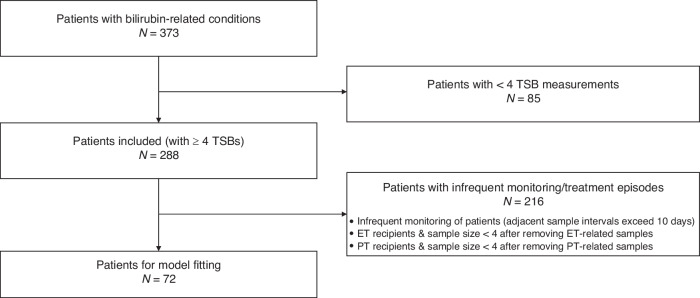
Study inclusion criteria. TSB total serum bilirubin, ET exchange transfusion, PT phototherapy.

The study inclusion is shown in the flowchart in Fig. 1. A total of 373 eligible subjects contributed 2208 TSB measurements during follow-ups. Of these, 85 infants with fewer than 4 measurements were excluded in the first stage. The remaining 288 infants, born between 24^2/7^ and 31^6/7^ weeks’ gestation, were thus included, aggregating 2011 TSB measurements in total. Moreover, 32 infants were monitored infrequently, 3 were exchange transfusion (ET) recipients under NICE guidelines and 273 were phototherapy recipients as documented in the database. Of these three exceptions, some patients overlapped, so a total of 216 patients were excluded leaving a subgroup of 72 patients for further analysis. A total of 421 TSB samples from the 72 patients were retained for modeling. In detail, the number of TSB measurements per infant is between 4 and 20 times with a median [IQR] of 5 [4, 6] samples. The initial bilirubin levels to be fit range from 128.6 to 210 µmol/L with a mean (SD) of 128.6 (31.1) µmol/L, and they were recorded at a median [IQR] PNA of 4.3 [2.0, 7.0] days. The last recorded TSB levels vary from 6 to 196 µmol/L with a mean (SD) of 70.8 (44.6) µmol/L, and they were acquired between 6.4 and 51.5 days after birth with a median [IQR] of 12.7 [9.7, 17.0] days.

Table 1 summarizes the general characteristics with descriptive statistics of the included 288 patients and the subgroup of 72 patients who were involved in patient-specific modeling. No significant difference was shown between the two populations.

**Table 1:** General characteristics of the included population and the subgroup used in patient-specific modeling.

	Characteristics	Included Patients *N* = 288	Modeled Patients *N* = 72	*P*-value
Initial conditions	Multiple pregnancy, *n* (%)	87 (30.2%)	15 (20.8%)	0.152
	Hypertension in pregnancy, *n* (%)	54 (18.8%)	18 (25.0%)	0.307
	Preterm labor, *n* (%)	192 (66.7%)	48 (66.7%)	1.000
	Chorioamnionitis, *n* (%)	19 (6.60%)	4 (5.56%)	0.957
	Corticosteroids, *n* (%)	268 (93.1%)	67 (93.1%)	1.000
	Delivery route, *n* (%)			0.506
	Vaginal delivery	121 (42.0%)	34 (47.2%)	
	C-section	167 (58.0%)	38 (52.8%)	
	GA at birth (weeks), mean (std); median	28.1 (1.74); 28.1	27.8 (1.72); 27.8	0.218
	Birth weight (g), mean (std); median	1082 (295); 1050	1009 (260); 960	0.051
	Birth weight Z-score^†^, mean (std); median	−0.03 (0.80); 0.05	−0.10 (0.87); 0.14	0.767
	Sex (male), *n* (%)	160 (55.6%)	36 (50.0%)	0.475
	Apgar (1-min score), median; [IQR]	6.00; [3.00, 8.00]	6.00; [2.00, 8.00]	0.715
	Intubation at birth, *n* (%)	62 (21.5%)	11 (15.3%)	0.310
	PDA on PNA = 4 days, *n* (%)	139 (48.3%)	40 (55.6%)	0.330
Outcomes	Neurologic impairment, *n* (%)	76 (26.4%)	17 (23.6%)	0.741
	Respiratory support stopped before 34 PMA weeks, *n* (%)	77 (26.7%)	15 (20.8%)	0.381
	Death, *n* (%)	16 (5.56%)	4 (5.56%)	1.000
	PNA at death (days), median; [IQR]	35.0; [19.5, 69.6]	43.3; [20.0, 98.4]	0.813
	PMA at death (weeks), median; [IQR]	33.2; [28.7, 36.6]	31.9; [27.8, 40.2]	0.813
	Interruption of follow-up, *n* (%)	2 (0.69%)	0 (0.00%)	1.000
	Length of follow-up (days), mean (std); median	30.0 (16.1); 27.2	32.9 (15.0); 28.3	0.106
	Phototherapy, *n* (%)	273 (94.8%)	62 (86.1%)	0.020

^†^ Z-scored birth weight based on gestational age according to Fenton’s 2013 preterm growth chart.^19^

Features are divided into initial conditions and outcomes according to the time of acquisition before or after PNA = 4 days. *GA* gestational age, *PDA* patent ductus arteriosus, *PNA* postnatal age, *PMA* postmenstrual age, *IQR* interquartile range.

### General TSB decay models

**Fig. 2:**
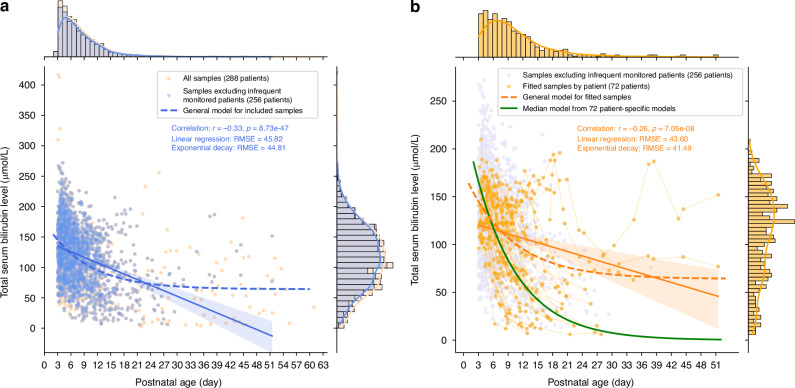
Total serum bilirubin (TSB) in μmol/L relative to postnatal age (PNA) in days for different populations and associated fitting models. **a** TSB measurements in µmol/L relative to PNA in days for the included population (*N* = 288) and general models of linear regression (solid blue line) and exponential decay (dashed blue curve). **b** TSB measurements in µmol/L relative to PNA in days for the fitted population (*N* = 72) and general models of linear regression (solid orange line) and exponential decay (dashed orange curve) and the median model from patient-specific models (solid green curve).

Figure 2 depicts the data distributions of TSB measurements (µmol/L) against corresponding PNA (days) for three population groups, with the histograms above and to the right providing the distribution of PNA and TSB levels, respectively. In Fig. 2a, the light-colored scatter points are all available TSB samples from the included population; the blue data points are from the 256 patients after excluding the infrequent monitored infants, presenting a negative correlation between TSB and PNA values. Two general models were fitted to these samples, and the exponential decay model achieved a marginally lower fitting error than the linear model. In the histograms, the long tail in the PNA distribution suggests that most repeated measurements were taken in the early periods after birth. The skewness of the bilirubin distribution indicates that though some infants present elevated bilirubin levels, the general population tends to have progressively lower bilirubin levels over time. Figure 2b shows the data distributions and general models for the 72 patients whose samples were used for patient-specific modeling and displays how the bilirubin levels of each patient changed over time. Based on this subgroup of measurements, excluding most of the unnatural values, the general models achieved similar trends and differences in RMSE as in Fig. 2a.

Overall, bilirubin levels tend to decrease as postnatal age increases. However, it is evident that the general models are not sufficient to accurately characterize the downward trend given significant large inter-individual variability. A great amount of data points notably deviated from the general curves, highlighting the necessity for more personalized modeling approaches.

### Model analyses: from patient-specific models to clinical outcomes

**Fig. 3:**
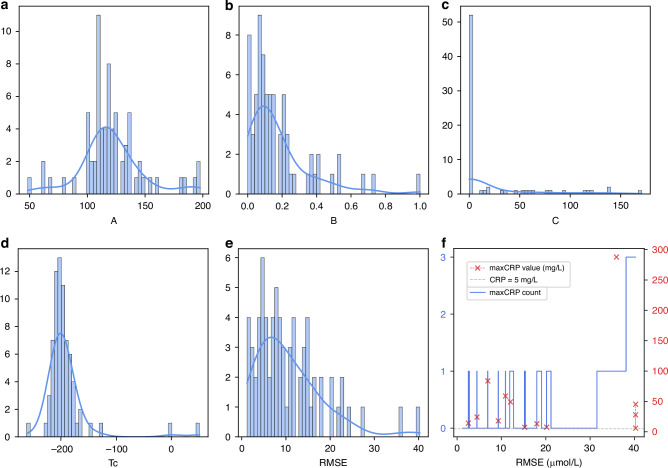
Histograms of parameters and fitting errors of patient-specific models among 72 patients. **a** Parameter $A$. **b** Parameter $B$. **c** Parameter $C$. **d** Parameter ${Tc}$. **e** RMSE. **f** Relationship between RMSE and counts of C-reactive protein (CRP) greater than 5 mg/L (blue step plot) and maximum CRP values (red scatter plot), respectively.

Patient-specific models were developed for 72 infants using selected TSB samples. For a given patient $i$, the function, as described in Eq. 3, was personalized by finding the optimal set of parameters ${A}_{i}$, ${B}_{i}$, ${C}_{i}$ and $T{c}_{i}$ that minimize the error between the model output and the observations. Histograms in Fig. 3a–d depict the distributions of these parameters. Within predefined boundaries, the distribution of parameter ${{{\boldsymbol{A}}}}$ have a mean (SD) of 120.90 (27.54), comfortably within the limits. Parameter ${{{\boldsymbol{B}}}}$ exhibits a median [IQR] of 0.11 [0.06, 0.21], clustering towards the lower limit. Parameter ${{{\boldsymbol{C}}}}$ extends up to 171, but nearly three-quarters of the values are concentrated at the lower boundary of 0. Parameter ${{{\boldsymbol{Tc}}}}$ ranges from −264.7 to −51.4, with a mean (SD) of −194.9 (42.0). The mean (SD) RMSE of 72 models is 10.62 (7.69), with a median [IQR] of 8.74 [4.89, 14.25], as shown in Fig. 3e. Figure 3f presents the counts and values of elevated CRP levels ( > 5 mg/L) across different RMSE values, considering only CRP results obtained post-phototherapy and before the last TSB measurements. Despite the limited sample size, a clear trend emerges: lower RMSE values correspond to fewer instances and lower maximum values of elevated CRP levels, whereas higher RMSE values are associated with an increase in both occurrences and higher maximum values of elevated CRP.

**Fig. 4:**
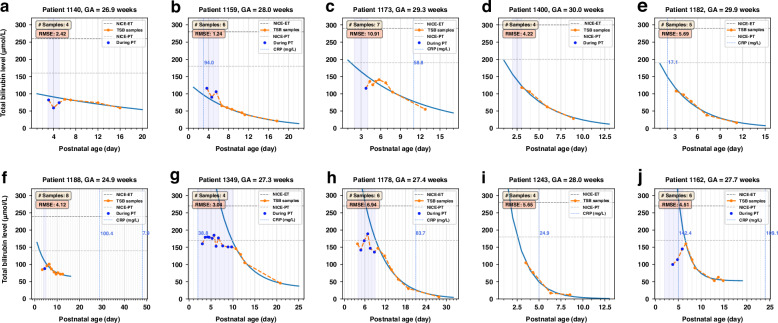
Ten representative instances of patient-specific models (blue solid curves) exhibiting various curve morphologies on the PNA-TSB plane, ordered by increasing rates of decay. **a** Patient 1140. **b** Patient 1159. **c** Patient 1173. **d** Patient 1400. **e** Patient 1182. **f** Patient 1188. **g** Patient 1349. **h** Patient 1178. **i** Patient 1243. **j** Patient 1162. The x-axes are postnatal age (PNA) in days; the y-axes are total serum bilirubin (TSB) in µmol/L. Annotations in light blue next to blue dashed vertical lines are C-reactive protein (CRP) values measured on corresponding PNAs. ET exchange transfusion, PT phototherapy, NEC necrotizing enterocolitis.

Figure 4 illustrates 10 instances of patient-specific well-fitted models exhibiting various curve morphologies ordered by increasing decay rates. Of these, models for Patients 1173, 1178, and 1243 shown in Figs. 4c, h, i were marked with significantly high CRP levels between two samples when their bilirubin levels were not frequently monitored, whereas Patients 1188 and 1162, as shown in Figs. 4f, j, experienced elevated CRP days after the cessation of TSB monitoring.

**Fig. 5:**
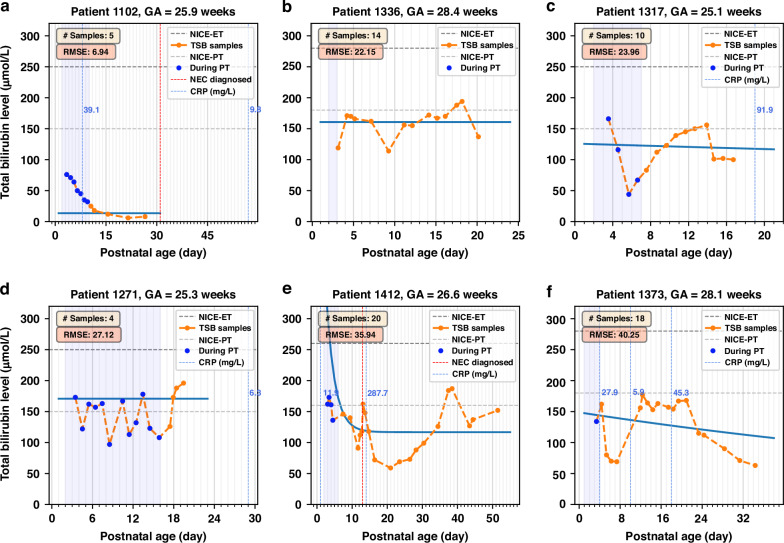
Patient-specific models with variability in TSB trends, modeling errors (RSME), and clinical outcomes. **a** Patient 1102. **b** Patient 1336. **c** Patient 1317. **d** Patient 1271. **e** Patient 1412. **f** Patient 1373. ET exchange transfusion, PT phototherapy, NEC necrotizing enterocolitis, CRP C-reactive protein.

Figure 5 presents instances of great variability in TSB trends, modeling errors, and clinical outcomes. Patient 1102 (Fig. 5a) was diagnosed with NEC on day 31, five days after the last bilirubin measurement. The remaining five models (Fig. 5b−f) depict infants who experienced recurrent hyperbilirubinemia after 2 weeks of life, with TSB levels exceeding the phototherapy thresholds recommended by the NICE guidelines. These models have the highest fitting errors evaluated by RMSE: four of them have the top four RMSEs and one model’s RMSE ranks seventh among the 72 patient-specific models. In detail, the TSB of Patient 1336 (Fig. 5b) fluctuated around the phototherapy threshold, peaking at 194 µmol/L around day 18. The patient had grade II-a enterocolitis from days 9 to 16, without inflammation or elevated CRP documented, corresponding to a rising TSB episode. Patient 1317 (Fig. 5c) shows a notable upward trend in TSB levels post-phototherapy, peaking on day 14. This infant had a persistent patent ductus arteriosus (PDA) that worsened progressively during the first two weeks and ended with surgical intervention on day 17. TSB monitoring ceased afterward, but the infant developed a late-onset infection on day 19 and died of septic shock the same day. Similarly, Patient 1271 (Fig. 5d) was born at 25.3 weeks of gestation and had a significant PDA from day 2, which was surgically closed on day 15. Figure 5e presents a complex case of Patient 1412, whose bilirubin levels dropped below 100 µmol/L post-phototherapy by day 11. However, a TSB rebound occurred on day 13, coinciding with an extreme CRP level of 287.7 mg/L and an NEC diagnosis. Then a second hyperbilirubinemia rebound was observed around day 38. Patient 1373 (Fig. 5f) underwent a notable decline in TSB levels after day 5, followed by a drastic rebound, with TSB fluctuating below the PT threshold. The infant developed cholestasis starting on day 5 (conjugated bilirubin 57 µmol/L), reaching a maximum on day 12 (conjugated bilirubin 173 µmol/L). Etiological investigations remained inconclusive, and the cholestasis eventually resolved. Subsequently, the infant developed grade II-a enterocolitis on day 17, with elevated CRP (45.3 mg/L) and a positive blood culture.

**Fig. 6:**
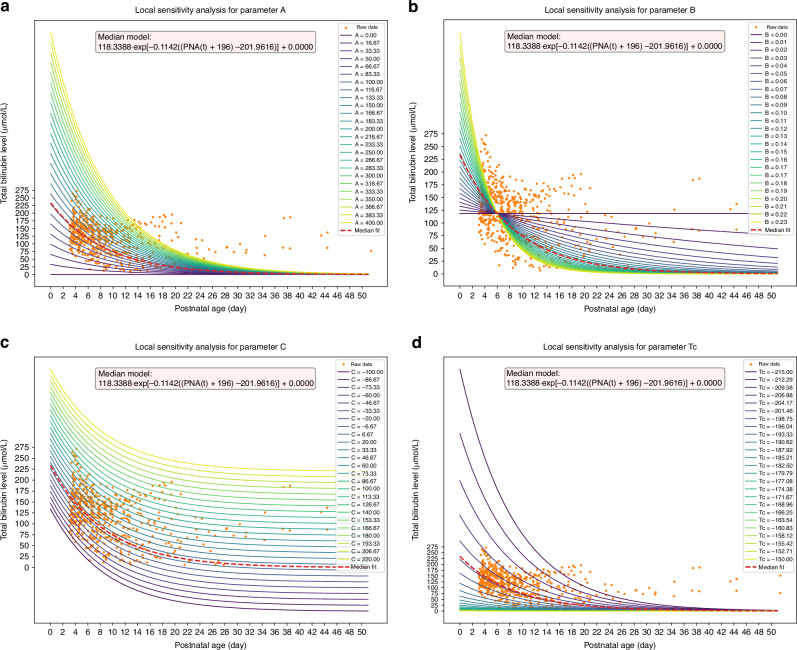
Local sensitivity analysis of parameter ${{{\boldsymbol{A}}}}$, ${{{\boldsymbol{B}}}}$, ${{{\boldsymbol{C}}}}$ and ${{{\boldsymbol{Tc}}}}$ in the median model. **a** Parameter ${{{\boldsymbol{A}}}}$. **b** Parameter ${{{\boldsymbol{B}}}}$. **c** Parameter ${{{\boldsymbol{C}}}}$. **d** Parameter ${{{\boldsymbol{Tc}}}}$.

Figure 6 illustrates the local sensitivity analysis of parameters ${{{\boldsymbol{A}}}}$, ${{{\boldsymbol{B}}}}$, ${{{\boldsymbol{C}}}}$ and ${{{\boldsymbol{Tc}}}}$ in the median model (illustrated by the red curve in Fig. 2b), revealing distinct patterns in their effects. In Fig. 6a, a series of curves represent models fitted with varying values of parameter ${{{\boldsymbol{A}}}}$, ranging from 0 to 400. As ${{{\boldsymbol{A}}}}$ increases, the curve shifts rightward and upward. Figure 6b shows the effect of parameter ${{{\boldsymbol{B}}}}$, the decay factor that controls the rate of decrease in the exponential function. The decay rate sharply rises as parameter ${{{\boldsymbol{B}}}}$ increases from 0 to 0.3. Figure 6c demonstrates how the curve is elevated as parameter ${{{\boldsymbol{C}}}}$ varies from −100 to 200. Lastly, Fig. 6d depicts the local sensitivity analysis of parameter ${{{\boldsymbol{Tc}}}}$, which controls the time shift within the support range of −215 to −150 days. As ${{{\boldsymbol{Tc}}}}$ becomes more negative, the curve shifts further rightward. The influence of **GA** in the model behaves similarly to ${{{\boldsymbol{Tc}}}}$, as both parameters occupy the same position in the proposed Eq. 3.

## Discussion

In this study, we proposed and quantitatively evaluated a patient-specific exponential decay model for characterizing the dynamics of TSB levels with age during the first weeks of life in a population of very preterm infants, as formalized in Eq. 3. To the best of our knowledge, this is the first study focusing on the natural history of TSB levels over such a long-term course in preterm infants born at 24–32 weeks of gestation.

The downward trend captured by the proposed exponential decay model is strongly associated with bilirubin metabolism. The normal metabolism of bilirubin can be summarized in five main steps^23^: production, uptake by the hepatocyte, conjugation, excretion into bile ducts, and delivery to the intestine. Defects or immaturity in any step of bilirubin metabolism can cause high concentrations of bilirubin in the blood, potentially resulting in hyperbilirubinemia. Preterm infants typically exhibit high bilirubin levels after birth, which is commonly referred to as physiological neonatal jaundice. Interventions, such as phototherapy, are often applied to manage TSB levels. With treatment, TSB levels usually fall back below critical thresholds and start to drop at a high rate of decline. As the infants age, their metabolic systems become more developed and refined, progressively achieving an equilibrium between bilirubin production and elimination. This process is characterized by a decelerating rate of decay and a relatively smooth tail, as depicted in the median evolutionary model (red curve in Fig. 2b). We propose that the evolution of these physiological phenomena can be effectively captured by specific model parameters.

The local sensitivity analysis of the median model elucidates the specific effects of each parameter on the model, detailing how changes in parameter values sculpt the model. Unlike the other three parameters that function independently, we explicitly decompose the time-shift term in the proposed model into two components: ${{{\boldsymbol{Tc}}}}$ and GA at birth. This decomposition enhances the model explainability. GA at birth is a key indicator of neonatal maturity, and its crucial impact has been consistently evidenced in the literature. Karen et al.^24^ reported that including GA in their assessment model for estimating the risk of significant hyperbilirubinemia in infants significantly improved predictive accuracy. Several reports have also noted that GA (and birth weight) plays a decisive role in the initiation and duration of phototherapy.^25,26^ The importance of GA is further corroborated by the NICE guidelines, which strictly differentiate treatment thresholds for phototherapy and exchange transfusion in neonates based on gestational age.^1^ In addition to GA, we explicitly incorporated another term, parameter ${{{\boldsymbol{Tc}}}}$, as part of the “time-shifter” in the model to account for varying maturity levels among neonates of the same gestational age. The results show that ${{{\boldsymbol{Tc}}}}$ values are all negative, this is because the values of GA are typically in hundreds of days, requiring ${{{\boldsymbol{Tc}}}}$ to act as a compensatory factor to bring the integrated time-shift term into the normal range.

By formulating a patient-specific function that describes postnatal age-based TSB levels as an exponential decay model, this study provides a more comprehensive description of the postnatal bilirubin decline in preterm infants with a gestational age of 24–32 weeks. On the one hand, the patient-specific model and its associated parameters reflect the similarities and variations of the natural development of bilirubin metabolism among the considered population. On the other hand, we demonstrated the predictive ability of the proposed model by monitoring deviations of actual bilirubin levels from the fitted natural bilirubin trends, with RMSE serving as one indicator to quantify these deviations. We illustrated this by annotating elevated CRP levels on the fitted models as shown in Figs. 4, 5. The discrepancies between measured TSB levels and the expected TSB trend identified by the proposed model could offer a valuable tool for disease detection, phenotyping, and prediction in the NICU. If a newly measured TSB level deviates from the expected decay, it could alert caregivers and prompt them to check for possible comorbidities such as infections, NEC, or other complications in the infant. A potential application could be a clinical decision support system based on explainable models for optimizing NICU monitoring and detecting high-risk events. Additionally, as shown in Figs. 4, 5, the lack of TSB measurements around elevated CRP levels exposes us to the risk of overlooking critical information. Therefore, it may be necessary to increase both the frequency and the duration of TSB monitoring. This would facilitate better observation of bilirubin dynamics and suspicious complications reflected by unnatural fluctuations in bilirubin. In clinical practice, TSB can be obtained through micro-sampling during other required biological monitoring without additional blood spoliation.

Nevertheless, this study has several limitations. First, the model-fitting phase excluded patients with fewer than four TSB measurements. We observed significant differences in 5 of the 13 initial conditions and 4 of the 8 outcomes between the included and excluded populations, as detailed in Supplementary Table S1. This exclusion might introduce selection bias and limit the generalization of the proposed model. Second, due to the limited sample size, we could only perform our analysis on the association between the proposed model and clinical events through qualitative assessments of a small number of cases. Future research could be dedicated to more systematic and extensive data collection and focus on quantitative analyses based on a richer database to evaluate the utility and performance of the model and the feasibility of using bilirubin levels as a potential indicator of high-risk clinical events in the NICU. Lastly, the CARESS-Premi clinical protocol only enrolled patient data from three days after birth. Consequently, the TSB evolution models proposed in this study lack information for the first three days. However, there is a large literature on the hourly trends of TSB in preterm infants within the first 72 or 96 h after birth,^14,15^ and what our research presented can plays a role as an extension and supplement to these previous studies, i.e., when combined, a comprehensive pattern of TSB development from birth to the several weeks postnatally could be portrayed.

## Conclusion

The proposed patient-specific exponential decay model proved efficient in characterizing the natural dynamics of TSB levels in preterm infants during the initial weeks post-birth. Through personalized modeling, the model and its parameters and fitting errors demonstrated indicative associations with clinical conditions, which is expected to provide valuable insights to healthcare professionals in the NICU to make informed therapeutic decisions. Moving forward, the potential integration of this explainable model into a model-based clinical decision support system holds significant promise for enhancing NICU monitoring optimization and facilitating high-risk event detection.

## Supplementary information

Supplementary Table S1

## Data Availability

The datasets generated and analyzed during the current study are not publicly available due to institutional ethics and privacy policies.
