# Supplementary material for: Model-based characterization of total serum bilirubin dynamics in preterm infants
Source: Pediatr Res. 2024 Nov 7;97(6):1873–81. doi: 10.1038/s41390-024-03644-z (PMC12122382; doi:10.1038/s41390-024-03644-z)
Supplement: Supplementary file 1 — Supplementary Table S1 [file 41390_2024_3644_MOESM1_ESM.docx]

Table S1. General characteristics of the included population and the excluded population (patients with fewer than four bilirubin measurements).

|  | Characteristics | No. of Records | Overall Population N=373 | Included Patients N=288 | Excluded Patients N=85 | P-value |
| --- | --- | --- | --- | --- | --- | --- |
| Initial conditions | Multiple pregnancy, n (%) | 373 | 104 (27.9%) | 87 (30.2%) | 17 (20.0%) | 0.088 |
|  | Hypertension in pregnancy, n (%) | 373 | 89 (23.9%) | 54 (18.8%) | 35 (41.2%) | < 0.001* |
|  | Preterm labor, n (%) | 373 | 225 (60.3%) | 192 (66.7%) | 33 (38.8%) | < 0.001* |
|  | Chorioamnionitis, n (%) | 373 | 22 (5.90%) | 19 (6.60%) | 3 (3.53%)) | 0.428 |
|  | Corticosteroids, n (%) | 373 | 349 (93.6%) | 268 (93.1%) | 81 (95.3%) | 0.626 |
|  | Delivery route, n (%) | 373 |  |  |  | 0.018 |
|  | Vaginal delivery |  | 144 (38.6%) | 121 (42.0%) | 23 (27.1%) |  |
|  | C-section |  | 229 (61.4%) | 167 (58.0%) | 62 (72.9%) |  |
|  | GA at birth (weeks), mean (std); median | 373 | 28.5 (1.82); 28.6 | 28.1 (1.74); 28.1 | 29.9 (1.42); 28.9 | < 0.001* |
|  | Birth weight (g), mean (std); median | 373 | 1133 (316); 1110 | 1082 (295); 1050 | 1306 (327); 1275 | < 0.001* |
|  | Birth weight Z-score^†^, mean (std); median | 373 | -0.05 (0.79); 0.02 | -0.03 (0.80); 0.05 | -0.11 (0.75); -0.14 | 0.211 |
|  | Sex (male), n (%) | 373 | 199 (53.4%) | 160 (55.6%) | 39 (45.9%) | 0.148 |
|  | Apgar (1 min score), median [IQR] | 370 | 6.00 [3.00, 8.00] | 6.00 [3.00, 8.00] | 6.00 [4.00, 8.00] | 0.963 |
|  | Intubation at birth, n (%) | 373 | 73 (19.6%) | 62 (21.5%) | 11 (12.9%) | 0.110 |
|  | PDA on PNA = 4 days, n (%) | 373 | 160 (42.9%) | 139 (48.3%) | 21 (24.7%) | < 0.001* |
| Outcome | Neurologic impairment, n (%) | 373 | 84 (22.5%) | 76 (26.4%) | 8 (9.41%) | 0.001* |
|  | Respiratory support stopped before 34 PMA weeks, n (%) | 373 | 119 (31.9%) | 77 (26.7%) | 42 (49.4%) | < 0.001* |
|  | Death, n (%) | 373 | 17 (4.56%) | 16 (5.56%) | 1 (1.18%) | 0.160 |
|  | PNA at death (days), median [IQR] | 17 | 29.5 [19.1, 66.0] | 35.0 [19.5, 69.6] | 15.4 [15.4, 15.4] | 0.353 |
|  | PMA at death (weeks), median [IQR] | 17 | 32.4 [28.8, 36.0] | 33.2 [28.7, 36.6] | 30.6 [30.6, 30.6] | 0.941 |
|  | Interruption of follow-up, n (%) | 373 | 2 (0.54%) | 2 (0.69%) | 0 (0.00%) | 1.000 |
|  | Length of follow-up (days), mean (std); median | 372 | 26.1 (16.8); 23.4 | 30.0 (16.1); 27.2 | 13.2 (10.7); 8.42 | < 0.001* |
|  | Phototherapy, n (%) | 373 | 344 (92.2%) | 273 (94.8%) | 71 (83.5%) | 0.001* |

* Statistical significance between included and excluded populations (*p* < 0.0025 after Bonferroni correction).

^†^ Z-scored birth weight based on gestational age according to Fenton's 2013 preterm growth chart ^19^.

*GA*: gestational age. *PDA*: patent ductus arteriosus. *PNA*: postnatal age. *PMA*: postmenstrual age. *IQR*: interquartile range.
